# Machine Learning Approaches to Assess Soil Microbiome Dynamics and Bio‐Sustainability

**DOI:** 10.1111/ppl.70719

**Published:** 2026-01-05

**Authors:** Roberta Pace, Maurilia M. Monti, Salvatore Cuomo, Antonio Affinito, Michelina Ruocco

**Affiliations:** ^1^ Department of Biology University of the Study of Naples “Federico II” Naples Italy; ^2^ Institute of Sustainable Plant Protection of the National Research Council (IPSP—CNR)—Portici Naples Italy; ^3^ Department of Mathematics and Applications “Renato Caccioppoli” University of the Study of Naples “Federico II” Naples Italy; ^4^ EVJA s.r.l. Naples Italy

**Keywords:** bio‐sustainbability, high‐throughput sequencing, machine learning (ML), metagenomics, soil microbiota

## Abstract

Understanding soil microbiota dynamics is essential for enhancing bio‐sustainability in agriculture, yet the complexity of microbial communities hampers the prediction of their functional roles. Artificial intelligence (AI) and machine learning (ML) offer powerful tools to analyse high‐dimensional microbiome data generated by high‐throughput sequencing. Here, we apply unsupervised AI‐based algorithms to uncover microbial patterns that are not immediately recognisable but are crucial for characterising the biological status of agricultural soils. Soil samples were collected from a site in Northern Italy managed under four strategies: conventional farming without organic matter (C), with organic matter (C + O), with beneficial microorganisms but without organic matter (M), and with both beneficial microorganisms and organic matter (M + O). Metagenomic amplicon sequencing of the 16S ribosomal RNA (rRNA) gene and the internal transcribed spacer (ITS) region was used to profile bacterial and fungal communities. Principal component analysis (PCA), k‐means clustering, and t‐distributed stochastic neighbour embedding (t‐SNE) revealed coherent temporal trajectories in both datasets, with sampling time and crop presence emerging as dominant drivers of community assembly and only subtle compositional shifts attributable to treatments. Fungal communities exhibited higher plasticity and a stronger response to management than bacterial communities, which converged towards a stable oligotrophic core. Our findings highlight the complementary roles of fungal and bacterial guilds and show that unsupervised ML‐based workflows provide an effective framework to disentangle temporal and treatment effects in complex microbiome datasets. This exploratory study lays the groundwork for future predictive models aimed at identifying microbial indicators of soil biological status and supporting bio‐sustainable agronomic decisions.

## 1 Introduction

Soil microbiota encompasses a diverse community of microorganisms inhabiting the soil, including bacteria, fungi, viruses and algae. These communities are integral components of the soil ecosystem, present across all soil types, with their abundance, diversity and composition significantly variable. Microorganisms adjust their community structure and functions in response to specific environmental and physicochemical soil conditions and are present across various ecosystems, including agroecosystems, forests, grasslands and even deserts (He et al. 2023; Cole et al. 2024; Zong et al. 2024; Burrill et al. 2025). Soil microbiota is distributed throughout the soil profile, with its highest concentrations typically found in the rhizosphere (Omotayo and Babalola 2021), thanks to the roots releasing root exudates, representing a readily available source of carbon and energy for microorganisms. This enrichment creates a biological ‘hotspot’: microbes proliferate more rapidly and establish highly dense and functionally distinct communities (Kuzyakov and Razavi 2019). They are also present in bulk soil, within aggregates, and in soil pores. Notably, a single gram of soil can contain up to 10 billion microorganisms spanning thousands of distinct species (Pandey et al. 2024).

Soil should be regarded as a non‐renewable resource and a vital and dynamic ecosystem in which organisms and microorganisms interact in a bidirectional manner (Nannipieri 2020; Pedrinho et al. 2024). These interactions are particularly crucial, as they profoundly influence the multifunctionality of the soil ecosystem (Wang et al. 2024; Hossain et al. 2025). A healthy soil sustains essential ecosystem services, including water retention, nutrient cycling, carbon sequestration, and erosion control. Moreover, it plays a critical role in food production by providing nutrients, water and structural support necessary for plant growth (Kopittke et al. 2024). Given these considerations, a fundamental question emerges: how can soil health be effectively assessed? Assessing soil health successfully involves evaluating its biological, chemical and physical properties to determine how well it supports plants, animals and humans. Physical indicators, in particular soil texture, aggregate stability, porosity and bulk density, give information on structure, compaction and water dynamics that influence root growth, water retention and gas exchange. Chemical indicators, such as pH, cation exchange capacity, organic matter content, nutrient levels (N, P, K, micronutrients) and presence of contaminants, and electrical conductivity, reflect fertility, nutrient cycling and pollution status. But, as largely discussed in scientific literature, an effective assessment of soil health must encompass a detailed analysis of its biological components, with particular emphasis on the soil microbial community, which also plays a pivotal role in mediating the soil–plant–ecosystem relationship (Doran and Zeiss 2000; Ferris and Tuomisto 2015; Lehman et al. 2015; Fierer et al. 2021; Bhaduri et al. 2022). The structure and functionality of these microbial assemblages critically influence nutrient cycling by decomposing organic material, thereby releasing plant‐available nutrients and contributing to the formation of humus, which enhances soil water retention (Lehmann and Kleber 2015). They also mediate key biochemical processes such as oxidation, reduction, solubilisation and chelation, facilitating nutrient availability (Martinez et al. 2013). By producing extracellular substances, these microorganisms contribute to soil aggregation, strengthening soil structure and stability (Hartmann and Six 2023; Mueller et al. 2024). Additionally, soil microbiota establishes interactions with plants that promote growth and improve resistance to biotic and abiotic stresses (Hou et al. 2021; Omae and Tsuda 2022; Kapoor et al. 2024).

Advancements in molecular techniques, particularly metagenomics, have revolutionised the study of soil microbiota by enabling a comprehensive assessment of microbial communities. This approach allows the analysis of genetic material directly from soil samples, providing insights into microbial diversity and abundance (Taraboletti et al. 2023), without the need for microbiological culture and thus overcoming the limitations posed by unculturable microorganisms. By facilitating comparisons across different environmental conditions and agricultural treatments, metagenomics serves as a powerful tool for detecting shifts in microbial populations that directly impact soil health and ecosystem functionality (Masuda et al. 2024).

This study focuses on soil microbiota, particularly fungal and bacterial communities within an intensively managed greenhouse system cultivating *Valerianella locusta* (lamb's lettuce). In such baby‐leaf systems, short crop cycles, continuous re‐sowing and limited crop rotation create a highly dynamic environment, in which temporal succession and rhizosphere effects may overshadow the impact of agronomic treatments. Yet, the relative contribution of the sampling time, crop presence and management to soil microbial dynamics remains poorly understood in these contexts.

Here, we investigate soil microbial communities through DNA‐based profiling and advanced computational analyses. We use AI‐based exploratory tools, principal component analysis (PCA), k‐means clustering, and t‐distributed stochastic neighbour embedding (t‐SNE) to identify major patterns and discrete microbial ‘states’ across treatments and sampling times. This unsupervised ML framework is designed to disentangle temporal versus treatment effects, compare the responses of fungal and bacterial communities, and provide a structured basis for future predictive modelling.

The ultimate goal is to pave the way for a predictive model that leverages artificial intelligence (AI) approaches, including machine learning (ML) and deep learning (DL) algorithms, to identify microbial patterns contributing to soil fertility. The analysis of soil microbiome data through artificial intelligence (AI) involves several critical steps (Pace et al. 2025):

1. **Data collection**—Ensuring the accuracy and representativeness of soil samples.
2. **Pre‐processing and transformation**—Cleaning, standardising, and preparing data for computational analysis.
3. **AI model selection**—Identifying appropriate algorithms for predictive modelling.
4. **Training and validation**—Developing and refining the model using sample data to enhance reliability.
5. **Model performance evaluation**—Assessing the model's accuracy and applicability in predicting microbial community dynamics.

Following this framework, we structured our research into three main phases: data collection, data analysis (pre‐processing and transformation) and model implementation. In the present work, we report on the outcomes of the first two phases. Specifically, we address the following questions: (1) How do soil fungal and bacterial communities evolve over successive crop cycles of *V. locusta* ? (2) To what extent do the four agronomic treatments, including microbial inoculation and organic matter addition, alter these temporal trajectories? and (3) Do fungal and bacterial communities differ in response to the same management regime? By answering these questions, we aim to clarify the ecological dynamics of soil microbiota in greenhouse baby‐leaf production and to illustrate how unsupervised ML approaches can support soil health assessment under agronomic conditions.

## 2 Materials and Methods

### 2.1 Site Description and Treatments

Soil sampling and environmental data collection were conducted from January to September 2023 in a commercial farm belonging to the Producer Organisation (OP) ‘Sole&Rugiada’, located in Bagnolo Mella (Brescia, Lombardy, Italy). The farm specializes in greenhouse cultivation of baby leaf crops, and the study was carried out on *Valerianella locusta* .

According to the experimental design, four treatments were established: (1) conventional farming without organic matter (C), (2) conventional farming with the addition of organic matter (C + O), (3) conventional farming with the addition of beneficial microorganisms but without organic matter (M), and (4) conventional farming with the addition of both beneficial microorganisms and organic matter (M + O). The term ‘addition of beneficial microorganisms’ refers to the application, via irrigation, of the commercial product ‘RyzoPepUp’ by Samagri S.r.l. (Cava de' Tirreni, NA, Italy), which contains rhizosphere bacteria and *Trichoderma* spp. This formulation is designed to promote the development of both the root system and the aerial parts of the crop. The product is delivered in an organic matrix consisting of a non‐composted plant‐based soil improver. In this experiment, RyzoPepUp was applied at a commercial dose and timing recommended by the manufacturer. Consequently, the presence or absence of the microbial inoculant (treatments M and M + O vs. C and C + O) was treated as a categorical management factor, rather than as a graded continuous input signal. Treatments with microorganisms were applied throughout the sampling period, with one application per crop cycle.

### 2.2 Soil Sampling Scheme and Experimental Design

Two baseline samples of bare soil were collected, one from the plots designated for the microbial inoculum (M/M + O) and one from the control plots (C/C + O). At that stage, the plots had only been demarcated, and no treatments had yet been implemented. Subsequent soil samplings were made at the end of each crop cycle. Sampling time points are thus denoted t0, t1, t2, t3, t4 and t5, with t0 representing the shared baseline and t1–t5 the subsequent samplings.

**FIGURE 1:**
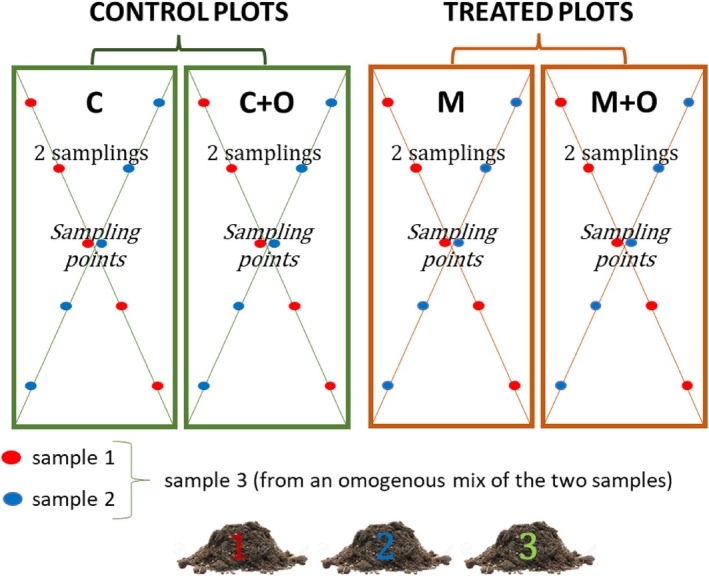
Schematic representation of the soil‐sampling design in the greenhouse experiment. In each of the four plots, two composite soil samples were collected by pooling cores at fixed positions along two diagonal transects (red and blue dots). A third soil sample was then obtained by mixing equal amounts (1:1) of the ‘blue’ and ‘red’ composites, yielding three samples (1–3) per plot.

For each treatment and time point, sampling points were arranged according to a standardised grid consisting of two diagonals across each plot, with five evenly spaced points per diagonal (Figure 1). Each diagonal yielded one composite soil sample, resulting in two samples per plot. From these two field replicates collected per treatment, a third composite sample was prepared by homogenisation to obtain a more representative biological replicate. As a result, three biological replicates were obtained for each treatment at each time point. All soil samples were collected in clean plastic bags, stored at 4°C, and processed within 1 week of collection. The sampling depth corresponded to the soil layer occupied by the root system, which, in the case of baby leaf crops, does not exceed 30 cm.

### 2.3 DNA Extraction and Amplicon Preparation

The biological characteristics of the soil samples were assessed through metagenomic analysis. Genomic DNA of bacteria and fungi was extracted from each soil sample and subsequently amplified in triplicate.

DNA extraction was carried out using the NucleoSpin Microbial DNA kit (Macherey‐Nagel), following the manufacturer's protocol for genomic DNA isolation from microbial communities. For bacterial community profiling, the universal primers 27F (forward: 5′‐AGAGTTTGATCCTGGCTCAG‐3′) and 1492R (reverse: 5′‐GCTTACCTTGTTACGACTT‐3′) (Frank et al. 2008) targeting the 16S rRNA gene were used. For fungal community analysis, the universal primers ITS1 (forward: 5′‐TCCGTAGCTGAACCTGCGG‐3′) and ITS4 (reverse: 5′‐TCCTCCGCTTATTGATATGC‐3′) (Mirhendi et al. 2007) targeting the ITS region were employed.

PCR amplification was carried out in a 50 μL reaction volume containing: 10 μL of 5X Colourless GoTaq Reaction Buffer with MgCl_2_ (Promega), 1 μL of forward primer (10 μM), 1 μL of reverse primer (10 μM), 1 μL of dNTP mix (10 mM each; Promega), 0.25 μL of GoTaq DNA Polymerase (5 U μL^−1^; Promega), 0.5 μL of DNA template (10 ng μ L^−1^), and 36.25 μL of sterile ultrapure water.

Thermal cycling was conducted using a Mastercycler Nexus X2 (Eppendorf) with the following conditions:

− For ITS amplification: initial denaturation at 95°C for 5 min; 35 cycles of 95°C for 30 s, 58°C for 30 s, 72°C for 50 s; final extension at 72°C for 10 min.
− For 16S amplification: initial denaturation at 95°C for 5 min; 35 cycles of 95°C for 30 s, 48°C for 30 s, 72°C for 90 s; final extension at 72°C for 10 min.

PCR products were visualised on a 1% agarose gel prepared in TAE 1% buffer and stained with SYBR Safe DNA Gel Stain (10%; Invitrogen, Thermo Fisher Scientific). A 100 bp DNA ladder (New England Biolabs Inc.) was used as a molecular size marker. Following electrophoresis, the amplicons were purified using the NucleoSpin Gel and PCR Clean‐up kit (Macherey‐Nagel) according to the manufacturer's protocol. The concentration of purified PCR products (ng μ L^−1^) was measured using both the NanoDrop One spectrophotometer (Thermo Fisher Scientific) and the Qubit Fluorometer (Invitrogen, Thermo Fisher Scientific) for improved quantification accuracy.

### 2.4 Oxford Nanopore Sequencing

Amplicon sequencing was performed by using Oxford Nanopore Technologies (ONT), which allows the analysis of near full‐length marker genes, improving taxonomic resolution compared to short‐read platforms. The three PCR replicates from each soil sample were pooled. Library preparation was performed using the Rapid Barcoding Kit 24 (SQK‐RBK114.24; Oxford Nanopore Technologies), and sequencing was carried out on a Flongle flow cell using the MinION platform, following the manufacturer's instructions.

### 2.5 Bioinformatic Workflow

Raw signals were processed with Guppy, the ONT software for basecalling and demultiplexing, retaining reads with a quality score ≥ Q7.

Subsequent bioinformatic processing was carried out using the EPI2ME platform (Oxford Nanopore Technologies). The workflow wf‐16S, which classified 16S/18S/ITS amplicons, was applied. This workflow includes read quality control, alignment against reference databases, and taxonomic classification. Relative abundances were calculated from the classified reads. Taxonomic assignments, performed via alignment against reference databases (e.g., GenBank, NCBI), were reported primarily at the genus level, with species‐level resolution considered when supported by sufficient read depth and classification confidence. The resulting abundance tables, reporting the number of reads per taxon per sample barcode, were exported in Excel format for downstream analyses.

### 2.6 Machine Learning Analytical Pipeline

Preliminary data cleaning and exploratory analysis were performed using Python (v3.11) as described in Supplementary (Table S1), with a pipeline that integrates several unsupervised machine learning techniques, namely principal component analysis (PCA), k‐means clustering and t‐distributed stochastic neighbour embedding (t‐SNE), to explore patterns and structure within complex microbial datasets and identify natural groupings among samples and potential microbial signatures associated with specific treatments or time points. For each dataset (ITS and 16S), we constructed a taxon (rows) × sample (columns) matrix of relative abundances, where each column corresponds to one treatment × time combination. These relative‐abundance matrices constitute the input to the ML pipeline, while treatment and sampling time were used only as metadata to colour and annotate ordinations and were not included as predictor variables. For the multivariate and machine‐learning analyses, the relative abundances from the three biological replicates were averaged so that each treatment × time combination was represented by a single composite profile. We chose this approach to reduce noise and focus on the main patterns associated with treatments and sampling times, while still accounting for within‐treatment variability at the level of data preprocessing. Metadata tables (one for ITS and one for 16S) have been included in Tables S4 and S5.

Principal component analysis (PCA) was applied to the centred and scaled relative abundance matrices to reduce dimensionality and summarise major gradients in community composition.

*K*‐means clustering was performed on the PCA scores to identify groups of samples with similar community structure. We explored values of *k* from 2 to 7 and evaluated clustering performance using the average silhouette score and inspection of the elbow in the within‐cluster sum of squares. For both ITS and 16S data, *k* = 3 provided the highest or near‐highest silhouette scores while avoiding very small, unstable clusters. Moreover, the three‐cluster solution yielded ecologically interpretable groups corresponding to early, intermediate and late successional stages, which were consistent across PCA and t‐SNE representations. Therefore, *k* = 3 was retained for all subsequent analyses.

To characterise the taxa associated with each of the three *K*‐means clusters, we combined the cluster assignments with the taxon relative‐abundance tables. For each dataset (ITS and 16S), we calculated the mean relative abundance of each taxon within each cluster and used these values to identify the taxa that were most abundant and characteristic of a given cluster. This procedure is descriptive and is intended to highlight taxa that typify each microbial ‘state’ identified by the clustering rather than to provide a formal statistical discriminant analysis.

To visualise non‐linear structure and local similarities in community composition, we applied t‐distributed Stochastic Neighbour Embedding (t‐SNE) to the same PCA scores used for *K*‐means. This approach preserves neighbourhood relationships and improves the separation of clusters in a two‐dimensional space. t‐SNE maps were coloured by treatment, sampling time, and *K*‐means cluster assignment to facilitate the joint interpretation of temporal dynamics and management effects.

These ML algorithms improved the interpretation of ecological dynamics and treatment effects, offering a data‐driven foundation for hypothesis generation. In particular, the *k*‐means algorithm indicates the number of clusters to be generated, determined through silhouette analysis, and is based on the average (centroid) of the points belonging to each cluster (Yuan and Yang 2019); while t‐SNE is an algorithm that refers to the use of the Student's t distribution in the low‐dimensional space to model similarities between points, and denotes the embedding method that aims to preserve neighbourhood relationships between data points when projecting them into a lower‐dimensional space, in a probabilistic (stochastic) manner (Jung et al. 2024).

## 3 Results

### 3.1 ITS Community Dynamics

#### 3.1.1 Multivariate Structure and Patterns

**FIGURE 2:**
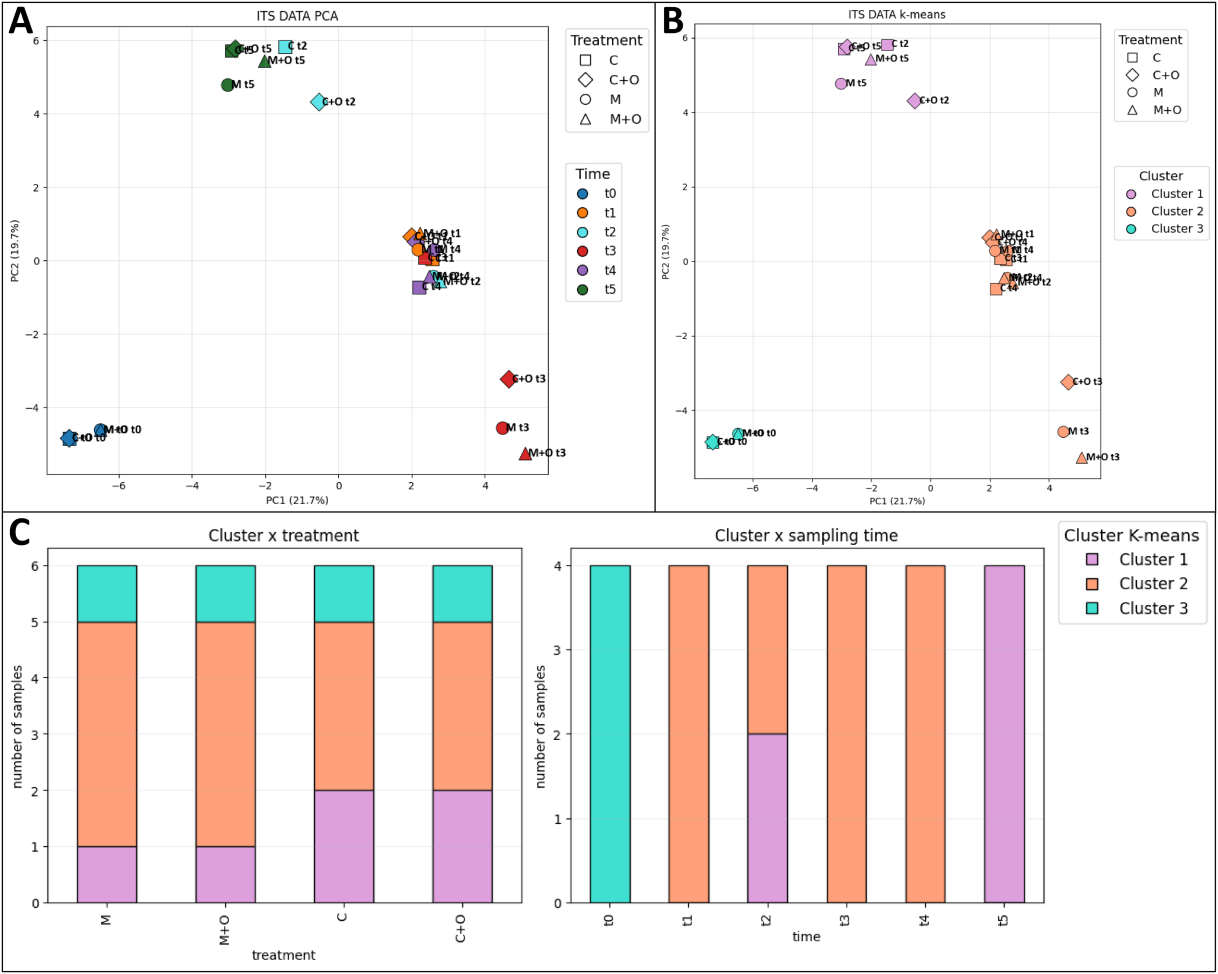
Multivariate structure and patterns of the ITS (fungal) community dataset. Principal Component Analysis (PCA) of the ITS (fungal) community dataset. (A) Ordination of samples along PC1 (21.7% of the variance) and PC2 (19.7%), with points coloured by sampling time (t0‐blue, t1‐orange, t2‐cyan, t3‐red, t4‐violet, t5‐dark green) and shaped by agronomic treatment (● M, ■ C, ▲ M + O, ◆ C + O); each point represents the mean community profile for one treatment × time combination (average of three biological replicates). (B) Same PCA ordination showing the three *K*‐means clusters (Cluster 1 = pink, Cluster 2 = salmon, Cluster 3 = turquoise) by colour, while point shapes again denote treatments. (C) Distribution of *K*‐means clusters in the ITS dataset, expressed as number of samples per cluster by treatment (left) and by sampling time (right).

The PCA of ITS sequencing data (PC1 = 21.7%, PC2 = 19.7%; cumulative variance ~41.4%) revealed a clear temporal separation of samples (Figure 2A). Baseline samples (t0) were clearly distinct from all subsequent time points, indicating a fungal community characteristic of bare soil before crop establishment. Samples collected from t1 to t4 occupied an intermediate region of the ordination space, whereas late‐stage samples (t5) were slightly shifted, suggesting further restructuring of the community towards the end of the cropping cycle. Treatment‐related differences were present but modest compared with the dominant temporal gradient.

*K*‐means clustering of PCA scores partitioned the ITS dataset into three clusters (Figure 2B). Cluster 3 contained all t0 samples and represented the initial community state. Cluster 2 encompassed the majority of samples from t1 to t4, while Cluster 1 comprised mainly late‐stage samples (t5) and a subset of mid‐cycle samples (t2) from C and C + O treatments.

Clustering analysis across all treatments (Figure 2C, left panel) reveals a strong predominance of Cluster 2 (salmon). The remaining clusters, Cluster 1 (pink) and Cluster 3 (turquoise), are less represented. Even if variability among treatments appears limited, and all three clusters occur across the four treatments, the proportions of the clusters seem to vary depending on the presence or absence of ‘RyzoPepUp’. Specifically, Cluster 2 dominates in M and M + O, whereas Cluster 1 gains greater importance in C and C + O.

In Figure 2C, right panel, clusters are shown in relation to sampling time. At t0, all samples fall within Cluster 3, suggesting an initial microbial community distinct from the subsequent phases. At t1, t3 and t4, all samples group into Cluster 2, which therefore dominates the intermediate stages of the cycle. At t2, samples are evenly divided between Cluster 1 and Cluster 2, indicating a transitional stage or increased community heterogeneity at this point, reflecting a new condition in specific samples. At the final stage (t5), all samples fall within Cluster 1, highlighting a marked reshaping of community composition compared to earlier phases.

**FIGURE 3:**
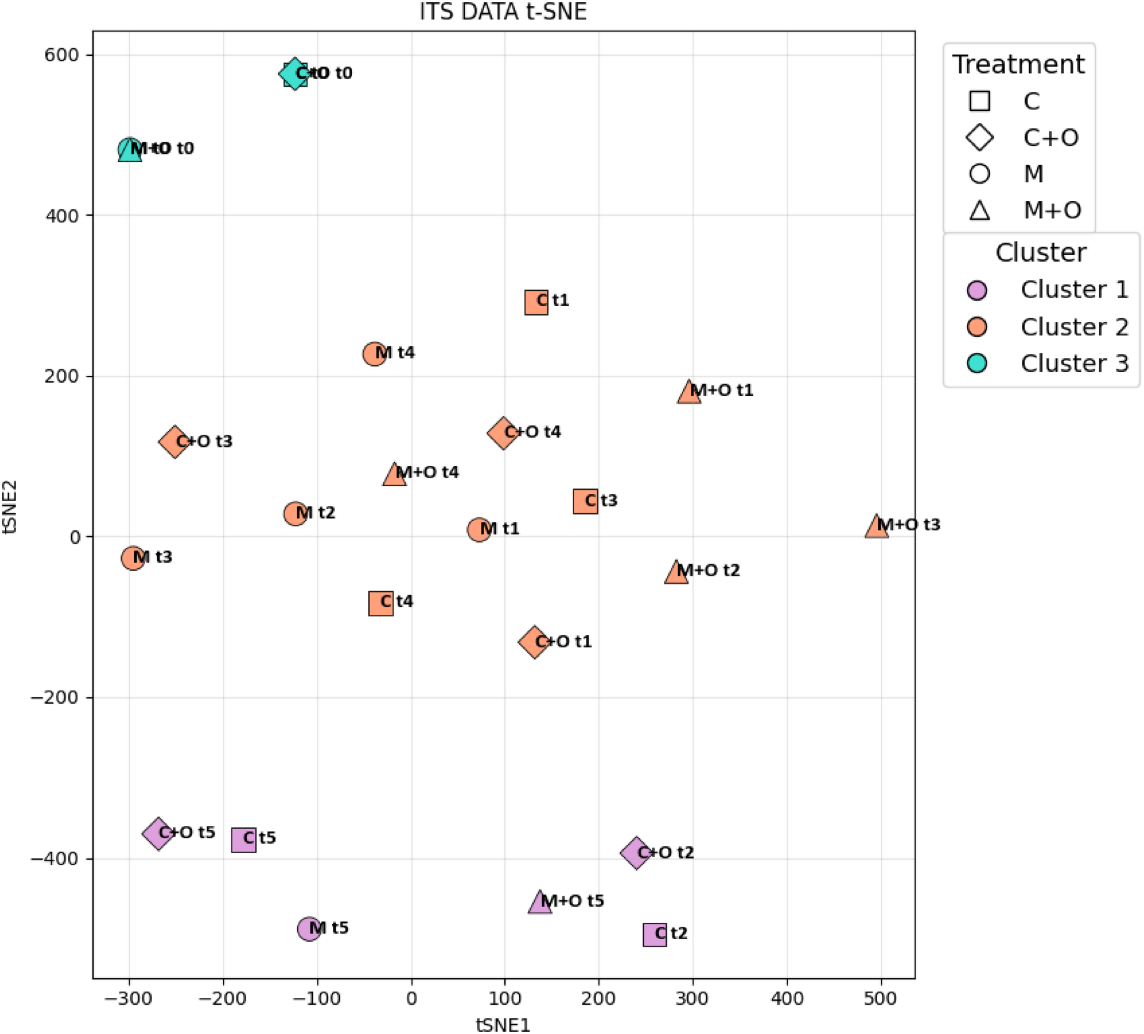
Non‐linear ordination of the ITS (fungal) community dataset. t‐distributed Stochastic Neighbour Embedding (t‐SNE) plot of ITS data showing the distribution of samples in the two‐dimensional t‐SNE space (tSNE1 vs. tSNE2). Each point represents a sample, with shapes indicating agronomic treatments (■ C, ◆ C + O, ● M, ▲ M + O) and colours corresponding to the three *K*‐means clusters previously identified (Cluster 1 = purple, Cluster 2 = orange, Cluster 3 = turquoise).

The t‐SNE analysis (Figure 3) preserved local similarities, making samples with similar features appear close together, thus revealing better cluster groupings. This analysis confirmed that sampling time, rather than treatment, was the main driver of fungal community composition. Early samples (t0) clustered separately, confirming the uniqueness of initial colonisers. Intermediate stages (t1–t4) were grouped predominantly in Cluster 2, indicating convergence of communities across treatments during the central phases of the experiment. At the final stage (t5), samples formed a distinct cluster, suggesting a shift towards treatment‐specific or late‐successional taxa. Only a subset of t2 samples grouped in Cluster 1 together with a sample of t5, highlighting a transient divergence. Overall, t‐SNE corroborated that temporal dynamics override treatment effects in shaping ITS‐based community structure.

#### 3.1.2 Cluster‐Level Taxonomic Signatures

The list of taxa that were most abundant within each fungal cluster, used to characterise their taxonomic signatures, is reported in Table S2. Below, we summarise the main taxa and functional groups that typify each cluster:

- Cluster 1 (C, C + O at t2 and M at t5) displayed a more heterogeneous composition. *Trichoderma* spp. co‐occurred with putative plant‐associated and pathogenic taxa (e.g., *Epicoccum* spp., *Paramacroventuria* spp.) and other functionally diverse fungi. This pattern suggests a late‐successional community in which beneficial and potentially harmful taxa interact under conditions of intensified root activity, resource competition and repeated cropping.
- Cluster 2 (which includes most samples from time points t1–t4) was characterised by a strong dominance of *Trichoderma* spp. together with fast‐growing saprophytes such as *Mortierella* spp. and *Linnemannia amoeboidea*. This assemblage likely represents an intermediate stage in which antagonistic fungi and opportunistic decomposers coexist under active rhizosphere influence and management interventions, while a background of saprotrophic fungi remains.
- Cluster 3 was clearly distinct (all samples at t0) and dominated by saprotrophic fungi typically associated with bulk‐soil organic matter turnover, including *Mortierella* spp., other Mucoromycota, and diverse decomposers. This profile is consistent with a decomposer‐driven community in a soil rich in plant residues and undergoing active carbon turnover and nutrient mineralisation prior to crop establishment.

These differences highlight shifts from an initial saprotrophic phase (Cluster 3, dominated by *Mortierella alpina*) to communities enriched in antagonistic taxa (Clusters 1, 2), likely reflecting contrasting soil conditions and crop‐associated dynamics and combining *Trichoderma* spp. with plant‐associated taxa. These results demonstrate that time is the dominant factor shaping ITS community structure, while treatments exert subtler but detectable effects.

**FIGURE 4:**
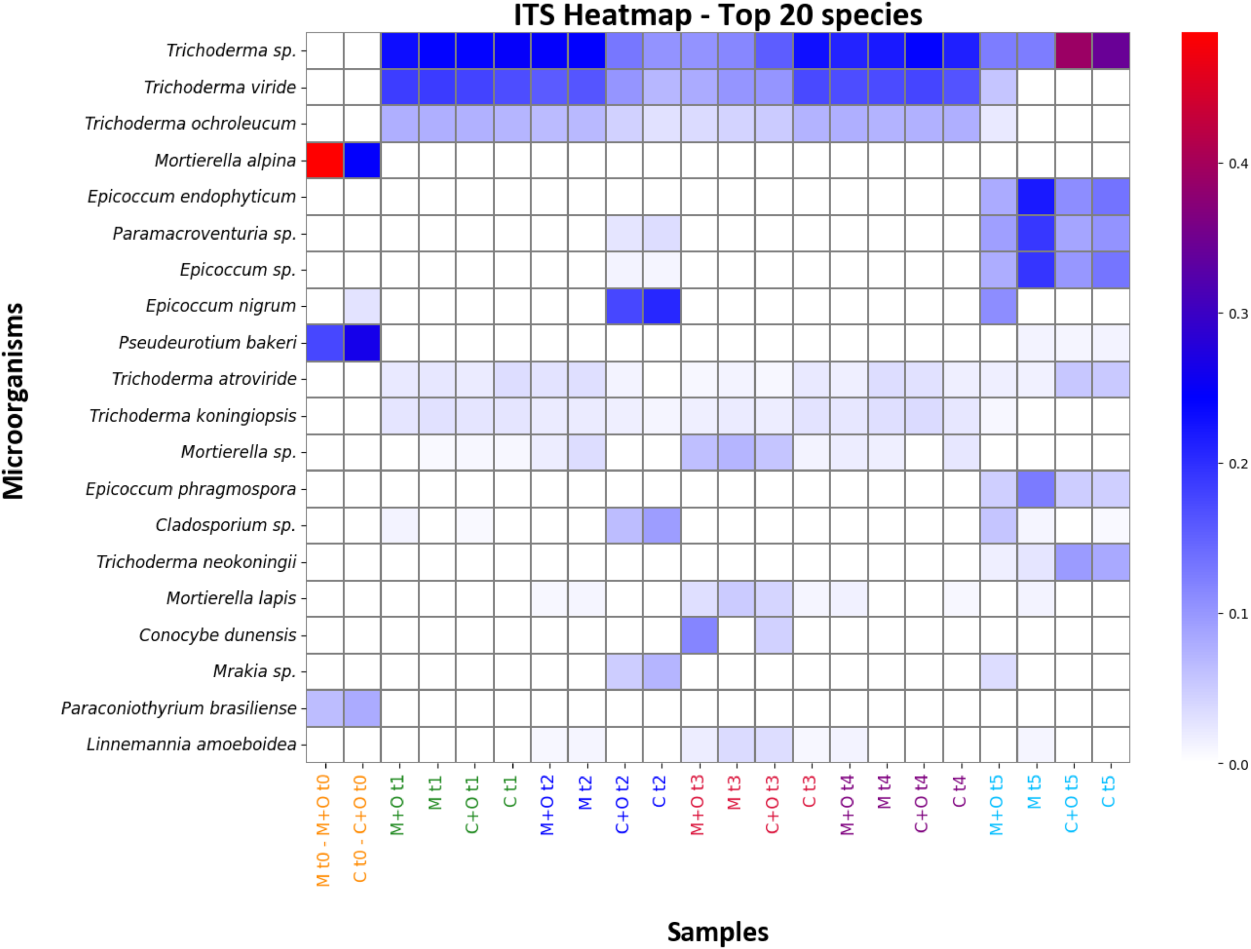
Heatmap of the 20 most abundant fungal species in the ITS dataset. Rows represent fungal species, while columns correspond to sample groups (treatments M, M + O, C and C + O) arranged along the temporal sequence of samplings (t0–t5); sample labels are colour‐coded by sampling time. Cell colours indicate relative abundance, ranging from white (absence or very low abundance) through blue (intermediate values) to red (highest abundance).

A heatmap of the top 20 fungal species across all samples (Figure 4) provided a concise overview of compositional patterns. The heatmap illustrates the relative abundance of microorganisms across samples and reveals clear differences in the distribution of fungal taxa among treatments and sampling times. Several taxa were detected at low abundance and appeared sporadically across samples, whereas others were consistently present and more abundant, shaping distinct community patterns. Rows correspond to fungal microorganisms identified through ITS sequencing, while columns represent sequenced samples, organised by treatment and time. The sidebar indicates relative abundance intensity, ranging from 0 (white) to increasing values in blue and up to the maximum in red. Notably, the t0 samples stand apart from subsequent ones; the samples collected between t1 and t4 display similar patterns, and those at t5 diverge again from the earlier time points, confirming what emerged from previous data analysis. Complete profiles (relative abundance of each taxon > 0.05) and details on taxa are reported in Table S4.

### 3.2 Bacterial (16S) Community Dynamics

#### 3.2.1 Multivariate Structure and Patterns

**FIGURE 5:**
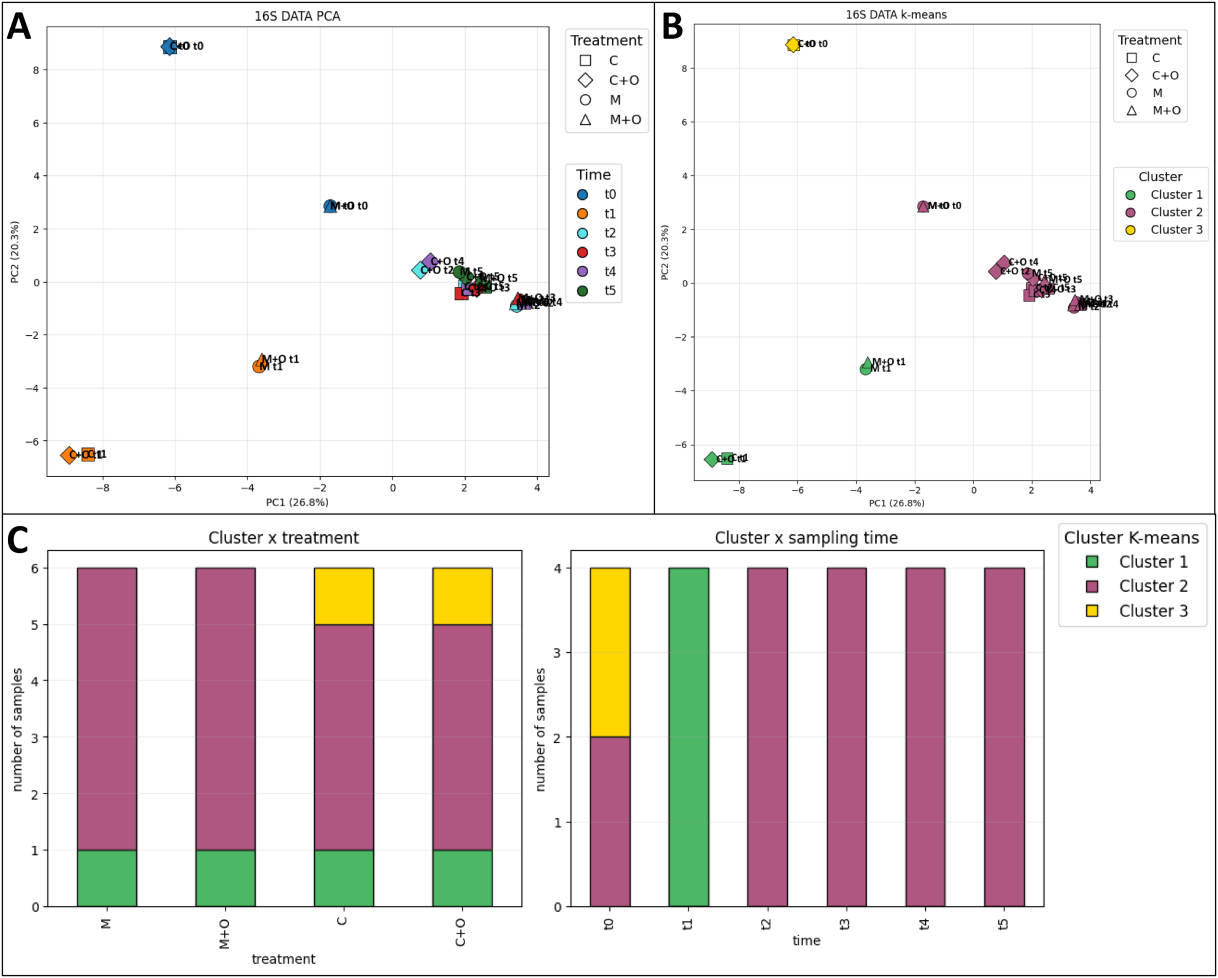
Multivariate structure and patterns of the 16S (bacterial) community dataset. Principal component analysis (PCA) of the 16S dataset. (A) Ordination of samples along PC1 (26.8% of the variance) and PC2 (20.3%), with points coloured by sampling time (t0–blue, t1–orange, t2–cyan, t3–red, t4–violet, t5–dark green) and shaped by agronomic treatment (● M, ■ C, ▲ M + O, ◆ C + O); each point represents the mean community profile for one treatment × time combination (average of three biological replicates). (B) Same PCA ordination showing the three *K*‐means clusters (Cluster 1 = green, Cluster 2 = purple, Cluster 3 = gold) by colour, while point shapes again denote treatments. (C) Distribution of *K*‐means clusters in the 16S dataset, expressed as number of samples per cluster by treatment (left) and by sampling time (right).

The PCA based on 16S data (PC1 = 26.8%, PC2 = 20.3%, cumulative variance ~47.1%), also revealed a marked effect of time on community composition (Figure 5A). Baseline samples (t0) formed two distinct groups reflecting the two baseline positions (C/C + O vs. M/M + O areas), both separated from subsequent stages. Samples from t2 to t5 clustered more closely together, indicating a convergence of bacterial communities as the cropping cycle progressed, whereas t1 samples occupied an intermediate but slightly more dispersed position.

*K*‐means clustering of the PCA scores identified three clusters in the bacterial dataset (Figure 5B). Cluster 3 grouped the t0 samples from C and C + O, forming the most distinctive assemblage. Cluster 2 included the t0 samples from M and M + O together with almost all samples from t2 to t5 across treatments, representing a stable core community. Cluster 1 comprised primarily t1 samples, reflecting a transient, early‐stage configuration.

Cluster 1 and Cluster 2 are present in all the treatments (Figure 5C, left panel), with Cluster 2 (purple) largely predominant. Cluster 3 (yellow) is only present in C and C + O treatments. This indicates that microbial composition does not markedly differ among treatments. A clear temporal pattern emerges (Figure 5C, right panel): at the initial time t0, samples are split between Cluster 2 (purple) and Cluster 3 (yellow); at t1, all samples fall exclusively into Cluster 1 (green), while from t2 to t5, all samples belong to Cluster 2 (purple).

This dynamic suggests that microbial variability is mainly driven by the temporal factor rather than by the treatment, with an initially heterogeneous community that progressively converges into a more uniform and stable profile as the cycle advances.

**FIGURE 6:**
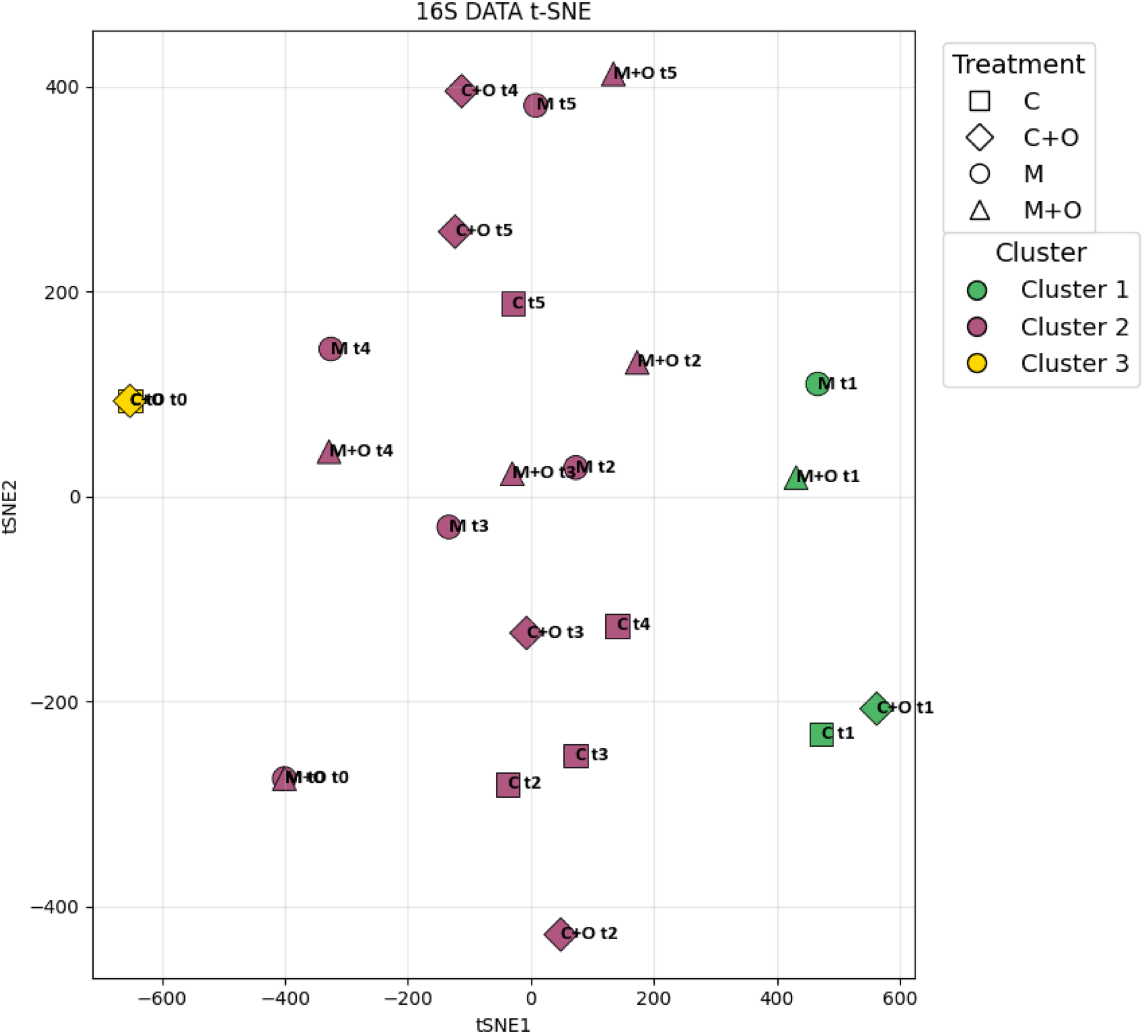
Non‐linear ordination of the 16S (bacterial) community dataset. t‐distributed stochastic neighbour embedding (t‐SNE) plot of 16S data showing the distribution of samples in the two‐dimensional t‐SNE space (tSNE1 vs. tSNE2). Each point represents a sample, with shapes indicating agronomic treatments (■ C, ◆ C + O, ● M, ▲ M + O) and colours corresponding to the three *K*‐means clusters identified (Cluster 1 = green, Cluster 2 = purple, Cluster 3 = gold).

The t‐SNE plot of 16S data (Figure 6) provided a complementary, non‐linear view. Baseline communities at t0 appeared as two clearly separated groups, t1 samples formed a small intermediate neighbourhood, and most samples from t2 to t5 converged in a central region, largely irrespective of treatment. This spatial arrangement confirmed that temporal dynamics were the predominant factor structuring bacterial communities, with treatments exerting only limited influence within this overall trajectory.

#### 3.2.2 Cluster‐Level Taxonomic Signatures

Analogously, the taxa that typified each bacterial cluster, based on their mean relative abundance within clusters, are summarised in Table S3. Cluster‐level taxonomic profiles showed that, in contrast to fungi, bacterial communities were dominated by a relatively stable oligotrophic core:

- Cluster 1 (mainly t1 samples) shared this core but was enriched in fast‐growing, disturbance‐tolerant and copiotrophic taxa, including some Bacillales and other stress‐tolerant lineages. This profile is consistent with a transient colonisation phase following crop establishment and early management operations, during which opportunistic bacteria temporarily increase in abundance before the community stabilises.
- Cluster 2, which included M/M + O t0 samples and almost all samples from t2 to t5, was structured around Acidobacteriota, Actinobacteriota and other taxa typical of low‐input, well‐aerated soils, together with diverse Proteobacteria (e.g., *Bradyrhizobium*, *Rhizobiales*‐affiliated taxa). This cluster reflects a community adapted to moderate resource availability and repeated cropping and likely underpins functional stability in the system.
- Cluster 3, comprising C and C + O t0 samples, was the most distinctive bacterial assemblage. It included a higher representation of taxa associated with wetter or more reduced microenvironments (e.g., denitrifiers and sulphur‐oxidising bacteria), together with organisms involved in nitrogen transformations and labile carbon turnover. This suggests that baseline conditions in the conventional plots before treatment application were characterised by higher moisture, micro‐anoxia and fresh organic inputs.

Together, these patterns suggest an ecological trajectory from heterogeneous initial communities (Clusters 2 and 3 at t0), through a transient colonisation phase (Cluster 1 at t1), towards a progressive stabilisation into a conserved and resilient core (Cluster 2 from t2 onwards). The analysis of cluster‐associated taxa further revealed a functional succession of microbial groups across the cropping cycle, corroborated by the temporal dynamics observed at each sampling point.

**FIGURE 7:**
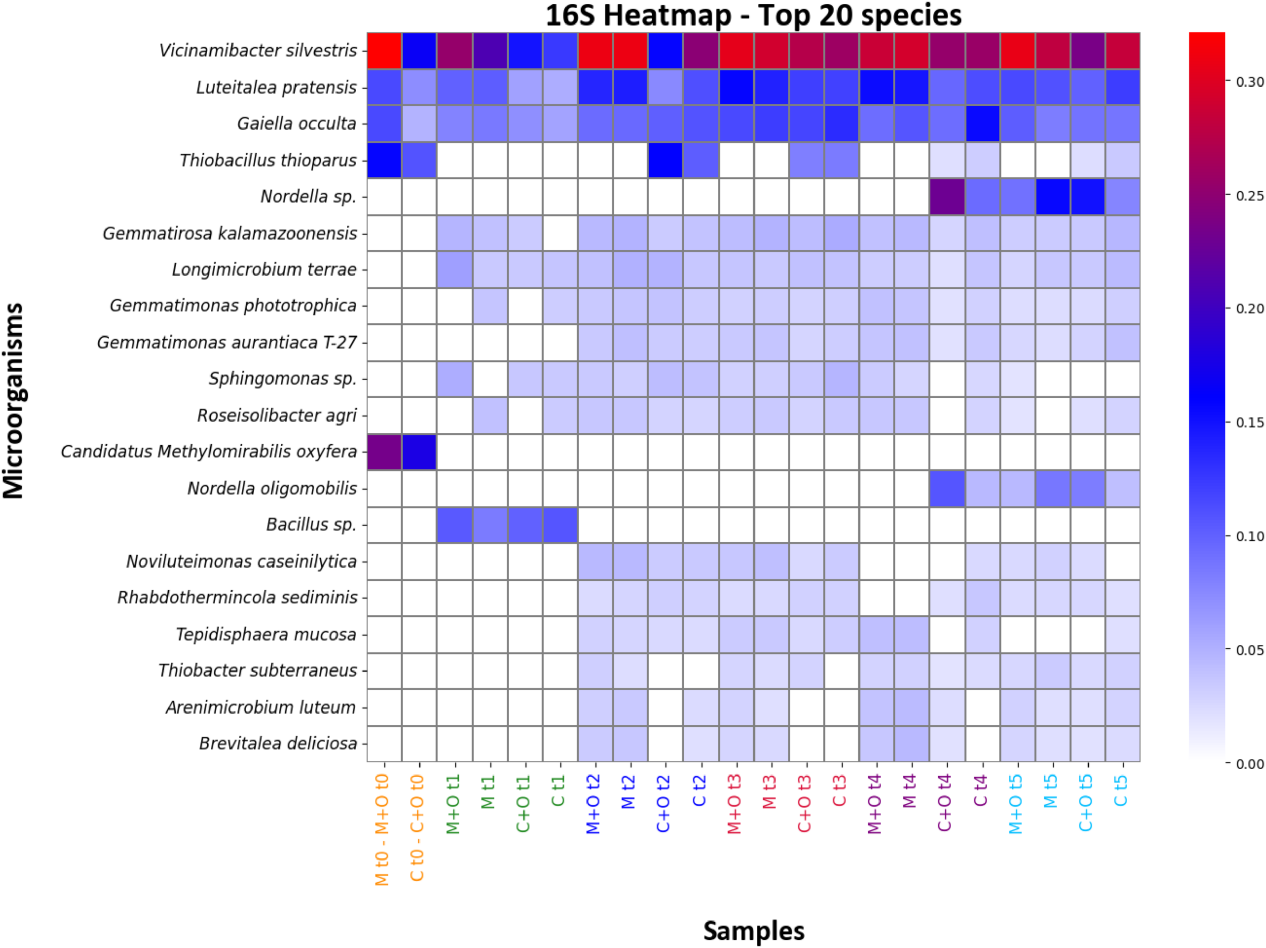
Heatmap of the 20 most abundant bacterial species in the 16S dataset. Rows represent bacterial species, while columns correspond to sample groups (treatments M, M + O, C and C + O) arranged along the temporal sequence of samplings (t0–t5); sample labels are colour‐coded by sampling time. Cell colours indicate relative abundance, ranging from white (absence or very low abundance) through blue (intermediate values) to red (highest abundance).

The heatmap of the top 20 bacterial species (Figure 7) summarised compositional changes across treatments and time. A small set of genera remained consistently abundant throughout the experiment, forming the core microbiome, while others showed transient peaks early in the crop cycle or slight shifts associated with particular treatments. Baseline samples (t0) exhibited more pronounced differences between conventional and microbial treatment areas, whereas samples from t2 to t5 were more similar across treatments, reflecting the convergence observed in multivariate analyses. Complete profiles (relative abundance of each taxon > 0.05) and details on taxa are reported in Table S5.

## 4 Discussion

This study combined amplicon sequencing of ITS and 16S rRNA genes with unsupervised machine learning to disentangle the effects of time, crop presence and agronomic treatments on soil fungal and bacterial communities in an intensively managed greenhouse system. The results indicate that temporal dynamics and the establishment of *Valerianella locusta* were the primary drivers of community assembly, whereas the four treatments produced only subtle compositional shifts. Fungal communities exhibited pronounced temporal restructuring and higher responsiveness to treatments, while bacterial communities converged towards a relatively stable core.

### 4.1 Temporal Dynamics and Crop Presence Dominate Over Treatment Effects

Our results provide complementary insights into the temporal and functional dynamics of soil microbial communities, integrating fungal (ITS) and bacterial (16S) profiles across treatments and sampling times. A consistent pattern emerges: time, rather than treatment, is the primary driver of microbial community assembly and succession.

At t0, when *V. locusta* was not yet established, microbial assemblages reflected bulk‐soil conditions, dominated by decomposer fungi (*Mortierella alpina*, *Pseudeurotium bakeri*) and metabolically specialised bacteria (*Candidatus Methylomirabilis oxyfera*, *Thiobacillus* spp.). These communities likely mirror the background soil legacy shaped by past management, organic residues, and resource availability in the absence of plant influence. Indeed, the genus *Mortierella* is well documented as a ubiquitous saprotroph enriched in bulk soils (Eberly et al. 2024), and early rhizosphere colonisation is often dominated by decomposers before giving way to more specialised taxa (Fracchia et al. 2024).

From t1 onwards, the establishment of *V. locusta* introduced the ‘rhizosphere effect,’ whereby root exudates and crop residues created novel ecological niches. This shift progressively favoured antagonistic and plant‐associated fungi (*Trichoderma* spp., *Epicoccum* spp.) and oligotrophic bacteria (Acidobacteriota, Actinobacteriota), consistent with previous studies (Windisch et al. 2021). The distinct *V. locusta* root bacteriome (García Méndez et al. 2023) may further contribute to these successional changes. Altogether, these dynamics point to a transition from bulk‐soil decomposer communities to rhizosphere‐shaped communities, progressively stabilising along the crop growth cycle.

Despite the application of organic matter and microbial inoculants, treatment effects on community composition were modest compared with temporal changes. Several factors may explain this limited differentiation. First, the greenhouse system is characterised by highly homogeneous management (irrigation, fertilisation, environmental control) and a relatively small spatial scale, which tends to reduce between‐plot variation. Second, the short duration of each crop cycle and the overlap between successive cycles may not allow sufficient time for treatment‐specific communities to develop and stabilise. Third, the added microorganisms and organic matter were applied according to commercial practise, which is designed primarily to enhance crop performance rather than to induce strong, lasting shifts in the entire microbiome. We note that the microbial inoculant was applied once per crop cycle, always using the same commercial formulation and recommended dose, so that our design effectively contrasted the presence versus absence of the inoculant as part of the overall management regime. Consequently, our inferences on inoculation effects are restricted to this specific product and application protocol, and the treatments are best interpreted as categorical management options rather than as quantitative levels of a continuous input signal. A dynamical‐systems approach aimed at building a robust predictive model would require varying inoculant type, concentration, and timing to generate richer input–output relationships and to characterise the underlying response functions more explicitly.

Under these conditions, the soil microbiota appears to be governed by strong deterministic forces linked to crop presence and overall management, while the treatments tested here act as secondary modifiers that slightly alter the prevalence of particular taxa or clusters, rather than restructuring the community as a whole. This does not exclude functional consequences of treatments; subtle shifts in key taxa may have disproportionate effects on nutrient cycling or plant health, but it suggests that such effects are embedded within a robust and recurrent temporal trajectory.

Taken together, these patterns indicate that our temporal resolution captures broad community states rather than fine‐grained dynamics. For fungi, t0 represents a distinct baseline state, samples from t1 to t4 form an intermediate rhizosphere‐influenced state, and t5 marks a late‐stage shift in community composition. For bacteria, t0 samples form distinct baseline groups, t1 represents a short‐lived transient configuration, and samples from t2 to t5 converge towards a common core community that remains relatively stable across treatments and late sampling times.

However, with six sampling times over the January–September period, we cannot resolve short‐term transients, such as rapid adjustments immediately after crop establishment, inoculant application or changes in environmental conditions. In particular, the transitions between t0 and t1 for fungi and between t1 and t2 for bacteria, as well as the detailed dynamics leading to the late‐season divergence observed at t5, remain undersampled. The divergence at t5 of the fungal communities may be linked to the cumulative effects of successive crop cycles and end‐of‐season changes in greenhouse climate and soil conditions, but our design does not allow us to pinpoint the exact drivers. Higher‐frequency sampling would be required to describe these transient phases in more detail.

### 4.2 Differential Plasticity of Fungal and Bacterial Communities

The functional transition observed reflects classic successional theory in soil microbial ecology: initial dominance by decomposer guilds gives way to biocontrol and plant‐associated taxa linked to plant health and rhizosphere interactions (Yu et al. 2025). At t0, fungal communities were dominated by saprotrophs (*Mortierella* spp., *Pseudeurotium* spp.) linked to organic matter turnover (Eberly et al. 2024), whereas later stages (t1–t5) were enriched in antagonistic fungi (*Trichoderma* spp., *Epicoccum* spp.) with known roles in plant growth promotion and pathogen suppression (Guzmán‐Guzmán et al. 2023; Yao et al. 2023).

On the bacterial side, the shift observed from opportunistic taxa and species associated with specific microenvironments (e.g., *Candidatus Methylomirabilis oxyfera*, *Pseudomonas* sp., *Thiobacillus* sp.) at t0 to oligotrophic and stress‐tolerant groups (Acidobacteriota, Actinobacteriota, Gemmatimonadota) from t1 onward is consistent with microbial life history theory (Zhang et al. 2025; Bao et al. 2021).

A central outcome of this study is the contrasting behaviour of fungi and bacteria.

The increased representation of *Trichoderma* spp. and other potential biocontrol fungi in mid‐ to late‐stage clusters is consistent with the idea that fungal communities can respond rapidly to changes in resource inputs, root exudation patterns and management, thereby modulating plant–soil feedbacks over relatively short time scales. Several studies have shown that soil fungi are particularly sensitive and fast responders to shifts in agricultural practises and organic matter inputs, often exhibiting greater or earlier changes than bacterial communities following modifications in tillage, fertilisation or mulching regimes (Zheng et al. 2018; Coller et al. 2021; Wipf et al. 2021; Zhang et al. 2024). Moreover, experimental work on rhizosphere interactions has demonstrated that root exudates can rapidly attract and stimulate specific fungal groups, including *Trichoderma* spp., and that these exudate‐driven shifts in fungal colonisation are associated with changes in plant defence activation and plant–soil feedback strength over a single growing season (Lombardi et al. 2018; Dutta et al. 2023; Steinauer et al. 2023). Together, these findings support our observation that fungal communities, and in particular biocontrol‐related taxa such as *Trichoderma*, can track short‐term changes in resource supply and rhizosphere conditions and thus act as dynamic modulators of plant–soil interactions.

In contrast, bacterial communities were dominated by a relatively stable oligotrophic core that persisted across treatments and time once the initial transient phase had passed. This pattern is consistent with the view that soil bacterial assemblages often contain a large fraction of slow‐growing, oligotrophic taxa (e.g., Acidobacteriota, many Actinobacteria) that form a persistent backbone of the microbiome and support key ecosystem processes under a range of environmental conditions (Tecon and Or 2017; Huusko et al. 2024; Jaeger et al. 2024). Such persistent cores are thought to exhibit a high degree of functional redundancy and niche complementarity, whereby multiple bacterial taxa perform overlapping roles in carbon and nutrient cycling, thereby buffering soil functions against species turnover and environmental fluctuations (Yin et al. 2000; Shade et al. 2012; Miki et al. 2014; Sauma‐Sánchez et al. 2024).

These differences underscore the complementary roles of fungi and bacteria in managed soils: fungi as more plastic responders that track changes in plant inputs and management, and bacteria as a resilient backbone that supports stable ecosystem processes. Recognising this complementarity is essential for designing management strategies that aim to harness the microbiome for soil health and crop productivity.

Because the present study is restricted to one greenhouse system, one crop species and a single period, these fungal–bacterial behaviours should be interpreted as site‐specific and hypothesis‐generating rather than universally generalisable. However, the information obtained provides a valuable baseline and reference framework for subsequent studies. Future work across multiple sites, years and crop types will be needed to assess how general these temporal trajectories and differences in plasticity between fungi and bacteria are under contrasting soil, crop and climate conditions.

### 4.3 Added Value of Unsupervised Machine Learning for Soil Microbiome Analysis

The unsupervised ML pipeline combining PCA, *K*‐means clustering and t‐SNE proved effective in reducing the complexity of high‐dimensional microbiome data, defining discrete microbial states and visualising temporal trajectories that are not readily captured by conventional diversity metrics alone. Dimensionality‐reduction techniques, such as PCA, are widely used in microbiome research to summarise major compositional gradients, while non‐linear embeddings, such as t‐SNE, can better reveal local neighbourhood structure and subtle transitions among communities. Recent reviews have highlighted the growing use of such unsupervised methods, including PCA, *K*‐means and t‐SNE, as core tools for exploratory analysis and feature extraction in microbiome and other omics datasets (Hernández Medina et al. 2022; Abavisani et al. 2024; Zhou and Zhao 2025). In line with these recommendations, our combined use of these techniques allowed us to collapse hundreds of taxa into a small number of ecologically interpretable axes, partition samples into recurrent microbial states associated with specific temporal phases, and visualise non‐linear trajectories from baseline to late‐cycle communities. These dynamics are not readily captured by conventional diversity metrics alone. Traditional α‐diversity indices (e.g., richness, Shannon) compress each community into a single value, so communities with markedly different taxonomic and functional profiles can display similar diversity scores (Finotello et al. 2018; Cassol et al. 2025). Likewise, standard *β*‐diversity measures and their ordinations (e.g., Bray–Curtis distances visualised by PCoA) summarise overall compositional dissimilarity but do not explicitly identify recurrent community states or non‐linear transitions over time, and their sensitivity depends on the chosen metric and normalisation strategy (Lemos et al. 2011; Kers and Saccenti 2022; Bars‐Cortina 2022). Importantly, the ML‐based clusters were ecologically interpretable: in fungi, they aligned with successional stages from decomposer‐dominated to rhizosphere‐influenced communities; in bacteria, they captured the progression from distinct baseline assemblies to a convergent core.

These observations are in line with recent reviews and applications showing that dimensionality reduction and clustering algorithms are increasingly used to extract latent structure from microbiome and other omics datasets, going beyond classic *α*/*β*‐diversity summaries and providing a pattern‐oriented foundation for downstream predictive modelling (Zhou and Gallins 2019; Shi et al. 2022; Armstrong et al. 2022; Abavisani et al. 2024).

## 5 Conclusions

By combining metagenomic amplicon sequencing of ITS and 16S rRNA genes with multivariate and unsupervised machine learning analyses, this study clarified how soil fungal and bacterial communities respond to time, crop presence, and agronomic treatments in an intensively managed greenhouse system for *Valerianella locusta* . Across both datasets, temporal dynamics and rhizosphere effects emerged as the dominant drivers of community assembly, whereas the four treatments (C, C + O, M, M + O) produced only modest compositional shifts.

Fungal communities exhibited marked plasticity, transitioning from saprotroph‐dominated assemblages at baseline to communities enriched in antagonistic and plant‐associated taxa at later stages. Bacterial communities, in contrast, rapidly converged towards a stable oligotrophic core dominated by Acidobacteriota, Actinobacteriota and other functionally redundant taxa, with only a brief transient phase following crop establishment. These contrasting responses highlight the complementary roles of fungi and bacteria in managed soils and the different degrees of stability and responsiveness they confer to the system.

The unsupervised ML analyses synthesised these patterns into a small number of recurrent microbial ‘states’ and temporal trajectories, offering an integrated view of microbiome dynamics that complements conventional diversity metrics without replacing them.

From an applied perspective, our findings indicate that, in intensive greenhouse baby‐leaf production, strategies aimed at improving soil bio‐sustainability should explicitly account for the strong and recurrent imprint of time and crop presence on the microbiome. Numerous studies have shown that plant development and rhizosphere processes can exert a stronger and more consistent influence on microbial community structure than individual management interventions, especially in systems with homogeneous inputs and short crop cycles (Schmidt et al. 2019; Wang et al. 2022; Zhao et al. 2019; Han et al. 2025). In this context, the modest treatment effects observed here suggest that management practises such as organic amendments and microbial inoculants may operate mainly by modulating specific guilds within a robust temporal trajectory, rather than by reshaping the community as a whole.

Our results also support the idea that fungal and bacterial communities contribute differently to soil functioning and respond with different degrees of plasticity and stability. Recent work has shown that fungal networks often display higher structural stability or stronger biogeographical structuring than bacterial networks, whereas bacteria can be more resilient and functionally redundant under environmental change (De Vries et al. 2018; Gschwend et al. 2021; Li et al. 2024; Wu et al. 2025; Fu et al. 2024). In our system, fungi behaved as more responsive levers of change, tracking shifts in resource inputs and crop development, while bacteria converged towards a stable oligotrophic core. This suggests that management strategies aiming to enhance soil bio‐sustainability in greenhouse baby‐leaf systems may be more effective if they explicitly target fungal functions, for example, by promoting beneficial saprotrophic and antagonistic taxa, while preserving the stability of the bacterial backbone that underpins key biogeochemical processes.

While the present work is exploratory and does not implement predictive models, the identification of recurrent microbial states and their associated taxa represents a crucial step towards AI‐assisted soil health assessment (Woodman and Mangoni 2023; Xu et al. 2020). In future work, these states and their discriminant taxa could be used as features in supervised ML models linking microbiome composition to soil functions, crop performance or resilience to stress, thereby advancing the development of predictive tools for bio‐sustainable management.

## Author Contributions

Conceptualisation and methodology: Roberta Pace, Maurilia M. Monti and Michelina Ruocco. Formal analysis and data curation: Roberta Pace. Visualisation, validation and supervision: Roberta Pace, Maurilia M. Monti and Michelina Ruocco. Writing – original draft preparation: Roberta Pace. Writing – review and editing: Roberta Pace, Maurilia M. Monti, Antonio Affinito, Salvatore Cuomo and Michelina Ruocco. Funding acquisition: Maurilia M. Monti and Michelina Ruocco. All authors have read and agreed to the published version of the manuscript.

## Funding

This study was conducted within the Agritech National Research Center and was funded by the European Union ‐ NextGenerationEU, under the Piano Nazionale di Ripresa e Resilienza (PNRR; Mission 4, Component 2, Investiment 1.4; Ministerial Decree d.d. 1032 of 17/06/2022, Project code CN00000022). The study was also carried out within the framework of Phen‐Italy, the Italian node of EMPHASIS (European Infrastructure for multi‐scale Plant Phenomics and Simulation for Food Security in a Changing Climate). The views and opinions expressed in this manuscript are those of the authors only and do not necessarily reflect those of the European Union or the European Commission; neither the European Union nor the European Commission can be held responsible for them.

## Conflicts of Interest

The authors declare no conflicts of interest.

## Supporting information


**Data S1:** ppl70719‐sup‐0001‐Supinfo1.pdf.

**Table S4:** ppl70719‐sup‐0002‐TableS4.xlsx.

**Table S5:** ppl70719‐sup‐0003‐TableS5.xlsx.

## Data Availability

The metagenomic sequencing data that support the findings of this study have not yet been deposited in a public repository but are available from the corresponding author upon reasonable request, and will be deposited in an appropriate public sequence repository no later than April 2026.
