# Supplementary material for: Machine Learning Approaches to Assess Soil Microbiome Dynamics and Bio‐Sustainability
Source: Physiol Plant. 2026 Jan 5;178(1):e70719. doi: 10.1111/ppl.70719 (PMC12766673; doi:10.1111/ppl.70719)
Supplement: Supplementary file 1 — Data S1: ppl70719‐sup‐0001‐Supinfo1.pdf. [file PPL-178-e70719-s003.pdf]

Machine learning approaches to assess soil microbiome dynamics and bio-sustainability

Roberta Pace[1,2,*], Maurilia M. Monti[2*], Salvatore Cuomo[3], Antonio Affinito[4], Michelina Ruocco[2]

[1]Department of Biology of the University of the study of Naples “Federico II” – Italy

[2]Institute of Sustainable Plant Protection of the National Research Council (IPSP – CNR) – Portici, Naples, Italy

[3]Department of Mathematics and Applications "Renato Caccioppoli" of the University of the study of Naples “Federico II” – Italy

[4] EVJA s.r.l. - Via Benedetto Brin, 63, 80142 Napoli NA

[*]Correspondence

Roberta Pace

E-mail: roberta.pace@unina.it

Mairilia M. Monti

E-mail: mauriliamaria.monti@cnr.it

SUPPLEMENTARY MATERIAL - S1

Preliminary data cleaning and exploratory analysis were performed using Python (v3.11) as described below:

- *pandas* (v2.0.3) and *numpy* (v1.25.0) for data handling and transformation;
- *scikit-learn* (v1.3.0) for dimensionality reduction and clustering;
- *scipy* (v1.11.1) for statistical calculations;
- *matplotlib* (v3.7.2) and *seaborn* (v0.12.2) for data visualization.

The analysis pipeline included:

- Data Preprocessing:
  Missing values (NaN) were replaced with zeros. Taxa with zero abundance across all samples were excluded;
- Taxonomic Aggregation and Cleaning:
  Identical taxa were merged by summing their values across samples;
- Replicate Aggregation and Normalization:
  Biological replicates (n=3) were averaged. Total Sum Scaling (TSS) was applied to obtain relative abundances;
- Filtering by Abundance:
  Taxon with relative abundance ≥ 0.5% in at least one sample were retained;
- Ordination and Clustering:
  Heatmaps were generated with hierarchical clustering based on Bray-Curtis dissimilarity. PCA was performed for ordination and dimensionality reduction;
- Advanced Pattern Detection:

K-means clustering was applied to identify sample groupings. The optimal number of clusters was first estimated using the silhouette score, and subsequently refined by considering their biological interpretability;

- Cluster-level taxonomic signatures:
  To characterise the taxa associated with each of the three K-means clusters, we combined the cluster assignments with the taxon relative-abundance tables. For each dataset (ITS and 16S), we calculated the mean relative abundance of each taxon within each cluster and used these values to identify the taxa that were most abundant and characteristic of a given cluster.
- Finally, t-SNE was employed for nonlinear dimensionality reduction and visual exploration of treatment-specific patterns.

SUPPLEMENTARY MATERIAL – S2

Cluster-level taxonomic signatures

Microorganisms	Cluster 1	Cluster 2	Cluster 3
Actinomucor elegans	0	0,00590626	0,016949153
Actinomucor elegans var. kuwaitensis	0,001179245	0	0
Ascomycota	0,009283036	0,003734107	0
Aureobasidium pullulans	0	0,000781746	0
Basidiomycota	0	0,005329599	0
Bolbitiaceae	0	0,006909918	0
Botryotrichum atrogriseum	0	0,000470905	0
Cladosporiaceae	0,010786071	0	0
Cladosporium halotolerans	0	0,001462079	0
Cladosporium sp.	0,038778297	0,001402539	0
Conocybe ceracea	0	0,001092732	0
Conocybe dunensis	0	0,011570276	0
Conocybe fuscimarginata	0	0,001211699	0
Conocybe incarnata	0	0,002813267	0
Conocybe sp.	0	0,004561471	0
Coprinellus radians	0	0,002764865	0
Coprinellus verrucispermus	0	0	0,031548541
Didymella sp.	0,004551071	0	0
Epicoccum brasiliense	0,004514645	0	0
Epicoccum endophyticum	0,090525931	0	0
Epicoccum nigrum	0,082116321	0	0,013317191
Epicoccum phragmospora	0,044785498	0	0
Epicoccum sp.	0,087522132	0	0
Hypocreaceae	0,01621748	0,070145852	0
Linnemannia amoeboidea	0,00166762	0,008864913	0
Linnemannia elongata	0	0	0,043377847
Linnemannia exigua	0	0,000555179	0,016620499
Linnemannia gamsii	0	0,002756488	0,015697138
Mortierella alpina	0	0	0,366792315
Mortierella globalpina	0,001429388	0,002786048	0
Mortierella lapis	0,001905851	0,012597342	0
Mortierella sp.	0	0,023903397	0
Mortierellaceae	0	0,001390809	0

Mrakia sp.	0,025600581	0	0
Nectriaceae	0	0,007751628	0
Neodidymelliopsis sp.	0,004532852	0	0
Nothophoma acaciae	0,007434235	0	0
Nothophoma sp.	0,00414891	0	0
Paraconiothyrium brasiliense	0	0	0,07180753
Paramacroventuria ribis	0,018236516	0	0
Paramacroventuria sp.	0,088597967	0	0
Pezizales	0	0,002174662	0
Plectosphaerella cucumerina	0	0,002776576	0
Podila minutissima	0	0	0,055936004
Preussia flanaganii	0	0	0,025608177
Pseudeurotium bakeri	0,005975457	0	0,219680557
Pseudeurotium hygrophilum	0	0,004093781	0,034780305
Pseudeurotium sp.	0	0,000831737	0
Pyrenochaetopsis decipiens	0	0	0,018159806
Rhizopus arrhizus	0	0,000822043	0,012003693
Saccharomyces cerevisiae	0,001155422	0,000976304	0
Sporormiaceae	0	0,002549321	0
Trichoderma asperellum	0	0,00658254	0
Trichoderma atroviride	0,024844071	0,021524598	0
Trichoderma austrokoningii	0	0,002537781	0
Trichoderma caribbaeum	0,008751894	0	0
Trichoderma dorotheae	0,0030677	0	0
Trichoderma gamsii	0,017052491	0	0
Trichoderma koningiopsis	0,005861405	0,024630221	0
Trichoderma neokoningii	0,036887054	0	0
Trichoderma ochroleucum	0,015960389	0,066938092	0
Trichoderma paucisporum	0	0,000685119	0
Trichoderma scalesiae	0,004283007	0	0
Trichoderma sp.	0,202682555	0,209919325	0
Trichoderma viride	0,037908075	0,155970037	0
Unclassified Fungi	0,083395715	0,316224746	0
Vacuiphoma ferulae	0,008361119	0	0
Westerdykella globosa	0	0	0,057721243

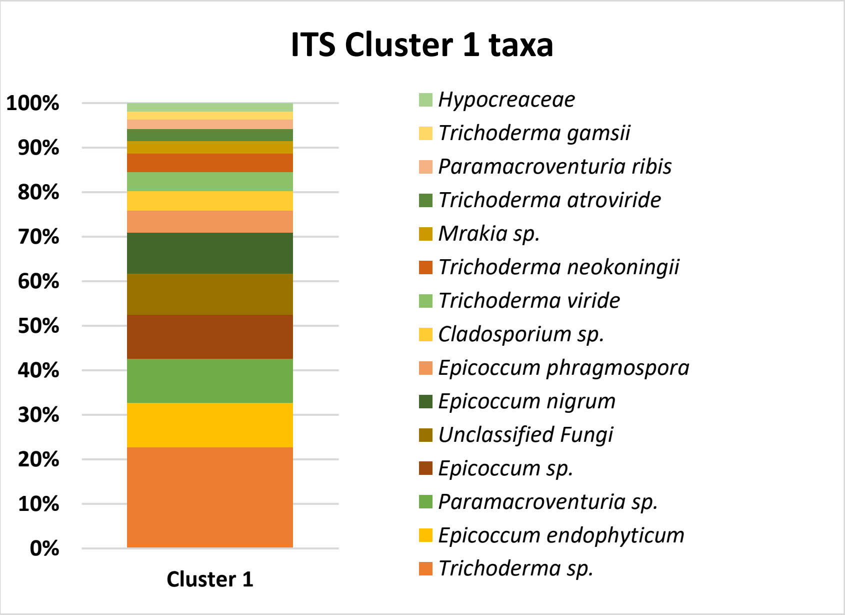

- Cluster 1 (C, C+O at t2 and M at t5) was characterized by a strong prevalence of *Trichoderma* spp. (including *T. viride, T. neokoningii, T. atroviride,* and *T. gamsii*) together with several species of *Epicoccum* and *Paramacroventuria*. This cluster is characterized by a dual structure: beneficial fungi (*Trichoderma* spp., *Epicoccum* spp.) co-occur with the phytopathogen *Paramacroventuria* sp. at high levels. Minor taxa such as *Cladosporium* spp., *Linnemannia amoeboidea* and *Mortierella* spp. are present but not dominant. This suggests a transitional or stressed community, where beneficial fungi and pathogens compete strongly. This may represent dynamic equilibrium under biotic stress, with potential implications for disease suppression vs. pathogen establishment.

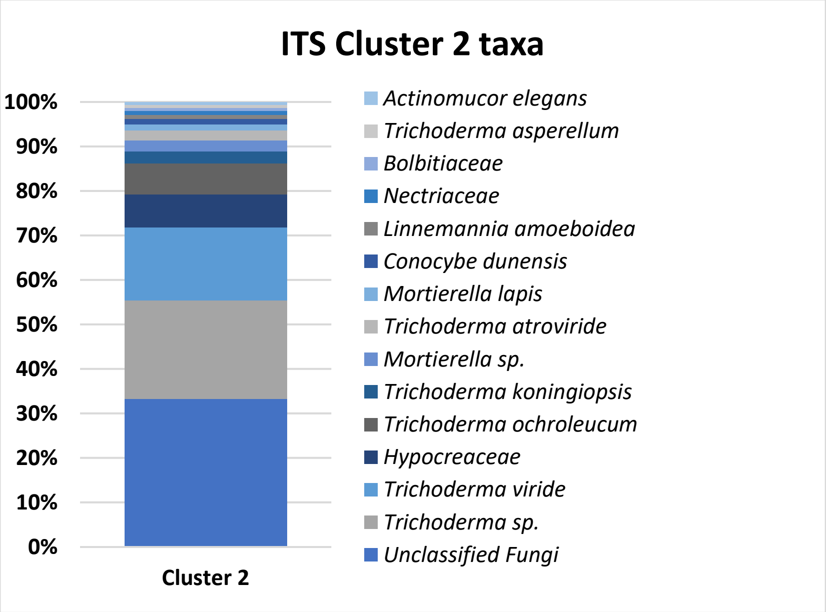

- Cluster 2 (which includes most samples from time points t1–t4) was overwhelmingly dominated by *Trichoderma* spp. (>45%), including *T. viride, T. ochroleucum, T. koningiopsis, T. atroviride,* and *T. asperellum*. It was further characterized by a high proportion of unclassified fungi and the co-occurrence of fast-growing saprophytes such as *Mortierella* spp., *Linnemannia amoeboidea,* and *Actinomucor elegans*. This assemblage suggests a transitional stage in which antagonistic taxa coexist with opportunistic decomposers, likely reflecting shifts in resource availability or disturbance. The additional presence of saprophytic

basidiomycetes (e.g., Bolbitiaceae, *Conocybe* spp., and *Coprinellus* spp.) points to active decomposition processes. Functionally, this profile aligns with rhizosphere or management conditions that promote biocontrol-oriented guilds, such as fertilization or microbial inoculation, while still sustaining a background of saprotrophic fungi.

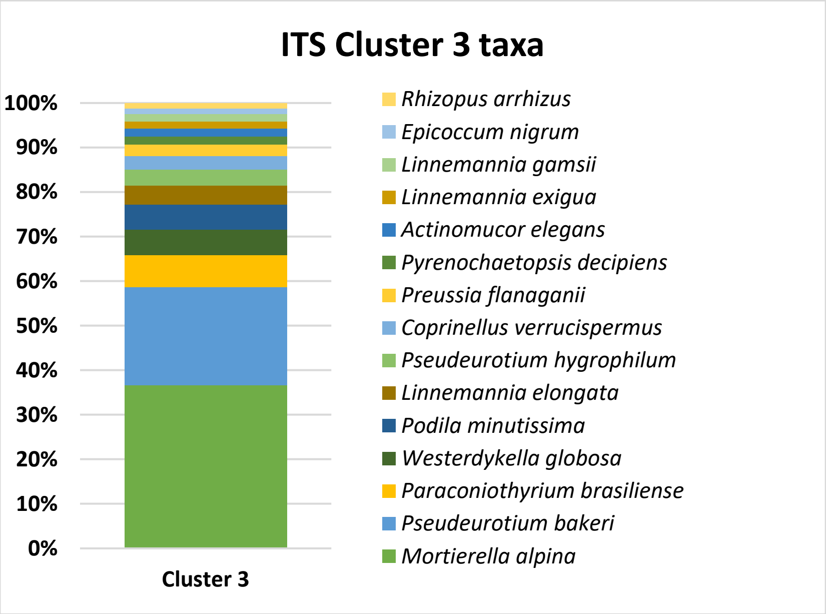

- Cluster 3 was clearly distinct (all samples at t0), dominated by *Mortierella alpina* and *Pseudeurotium bakeri*, alongside other saprophytic fungi (*Coprinellus verrucispermus*, *Linnemannia* spp., *Paraconiothyrium brasiliense*, *Podila minutissima*, *Westerdykella globosa*), pointing to a community strongly oriented towards organic matter decomposition and nutrient turnover. This may reflect a decomposer-driven cluster, suggesting an environment rich in organic residues and undergoing active carbon turnover and nutrient mineralization.

SUPPLEMENTARY MATERIAL – S3

Cluster-level taxonomic signatures

microorganisms	Cluster 1	Cluster 2	Cluster 3
Aciditerrimonas ferrireducens JCM 15389	0	0,002133474	0
Antarcticibacterium flavum	0,024353599	0	0
Arenimicrobium luteum	0	0,018917546	0
Bacillus badius	0	0	0,036363636
Bacillus sp.	0,098676659	0	0
Brevitalea deliciosa	0	0,018233334	0
Brevitalea sp.	0	0,002701503	0
Candidatus Methylomirabilis oxyfera	0	0,02603526	0,177777778
Candidatus Udaeobacter copiosus	0	0,006560066	0
Chujaibacter soli	0,027794085	0	0
Cognatiluteimonas lumbrici	0,020006277	0	0
Cytobacillus spp.	0,017771601	0	0
Gaiella occulta	0,07262389	0,106721181	0,048484848

Gemmatimonas aurantiaca T-27	0	0,028595058	0
Gemmatimonas phototrophica	0,017007006	0,026895734	0
Gemmatirosa kalamazoonensis	0,030084012	0,035964557	0
Geoalkalibacter ferrihydriticus	0	0,000907153	0
Limisphaera ngatamarikiensis	0	0,003057825	0
Longimicrobium terrae	0,041452627	0,032345426	0
Luteitalea pratensis	0,078557682	0,121499078	0,072727273
Mesobacillus subterraneus	0,007024996	0	0
Neobacillus cucumis	0,02003269	0	0
Neobacillus niacini	0	0	0,034343434
Nitrosospira multiformis	0	0,001609982	0
Nocardioides sp.	0,016355057	0	0
Nordella oligomobilis	0	0,022529959	0
Nordella sp.	0	0,04412057	0
Noviluteimonas caseinilytica	0	0,02185564	0
Novilysobacter spongiicola	0,016142384	0	0
Ohtaekwangia koreensis	0	0,006594398	0
Peribacillus asahii	0	0	0,094949495
Povalibacter uvarum	0	0,012555066	0
Priestia aryabhattai	0,05614783	0	0
Priestia aryabhattai B8W22	0,081034186	0	0
Priestia megaterium	0,051329887	0	0
Priestia sp.	0,018153031	0	0
Pseudomonas fluorescens	0	0	0,050505051
Pseudomonas putida	0	0	0,088888889
Pseudomonas sp.	0	0,001252208	0
Pseudomonas umsongensis	0	0	0,034343434
Rhabdothermincola sediminis	0	0,020382897	0
Roseisolibacter agri	0,017954807	0,023448848	0
Rossellomorea marisflavi	0,008827376	0	0
Salinimicrobium soli	0,047358045	0	0
Sphingomonas sediminicola	0	0,001212313	0
Sphingomonas sp.	0,030540284	0,021754013	0
Stenotrophobacter terrae	0	0	0,042424242
Tepidisphaera mucosa	0	0,020105097	0
Thermoanaerobaculum aquaticum	0	0,002178897	0
Thiobacillus denitrificans	0	0,001498178	0,044444444
Thiobacillus sp.	0	0,007582528	0
Thiobacillus thioparus	0	0,04718986	0,109090909
Thiobacillus thiophilus	0	0,010568425	0
Thiobacter subterraneus	0	0,019072218	0
Tumebacillus ginsengisoli	0,008291129	0	0
Vicinamibacter silvestris	0,183788805	0,277194615	0,165656566
Vicinamibacter sp.	0	0,006727093	0
Vreelandella sp.	0,008692053	0	0

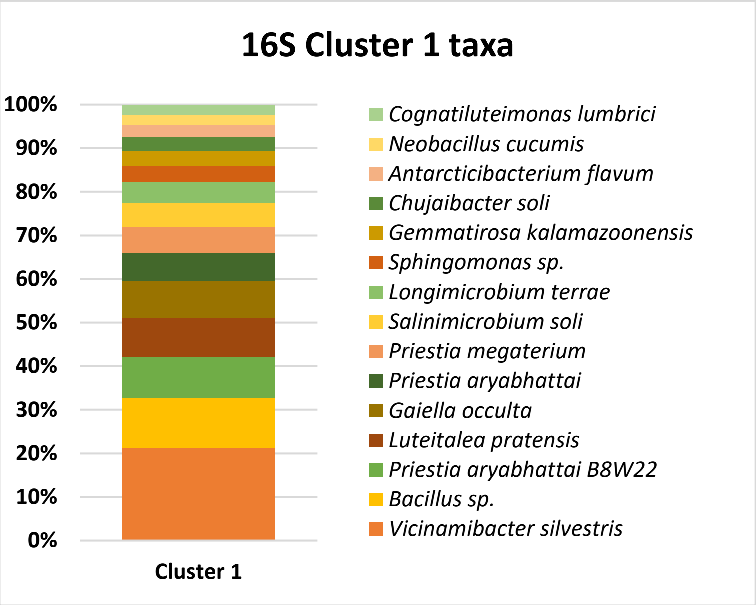

- Cluster 1 (t1 samples) in addition to the core taxa, shows a strong enrichment of Firmicutes/Bacillaceae (*Bacillus sp.*, *Priestia aryabhattai*, *P. megaterium*, *Neobacillus cucumis*) and versatile/tolerant taxa (*Salinimicrobium soli*, *Longimicrobium terrae*, *Sphingomonas sp.*, *Gemmatirosa kalamazoonensis*). This profile is consistent with more aerated conditions and moderate organic inputs: spore-formers suggest disturbance/drying cycles and persistence capacity; *Sphingomonas* points to the degradation of complex/aromatic compounds. The presence of *Salinimicrobium* also indicates neutral to alkaline soils with moderate osmotic stress.

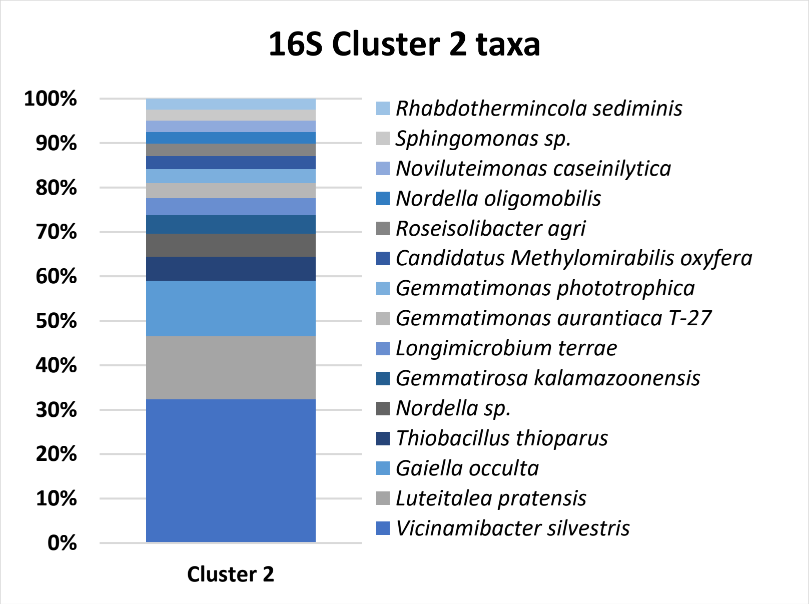

- Cluster 2 (M and M+O t0 e all samples from t2 to t5), alongside the shared core, includes *Thiobacillus thioparus* (thiosulfate/sulfur oxidation), members of Gemmatimonadota (*Gemmatirosa kalamazoonensis*, *Gemmatimonas phototrophica*, *G. aurantiaca*), *Nordella* spp., *Roseisolibacter agri*, *Noviluteimonas caseinilytica*, *Rhabdothermincola sediminis*, and *Sphingomonas*. Altogether, these indicate micro-niches with reduced sulfur compounds and the presence of Gemmatimonadota, often linked to drier/oxidized soils. Cluster 2 therefore appears intermediate/transitional: it maintains an oligotrophic backbone but integrates chemolithotrophs and taxa typically enriched under dry conditions or fluctuating moisture regimes.

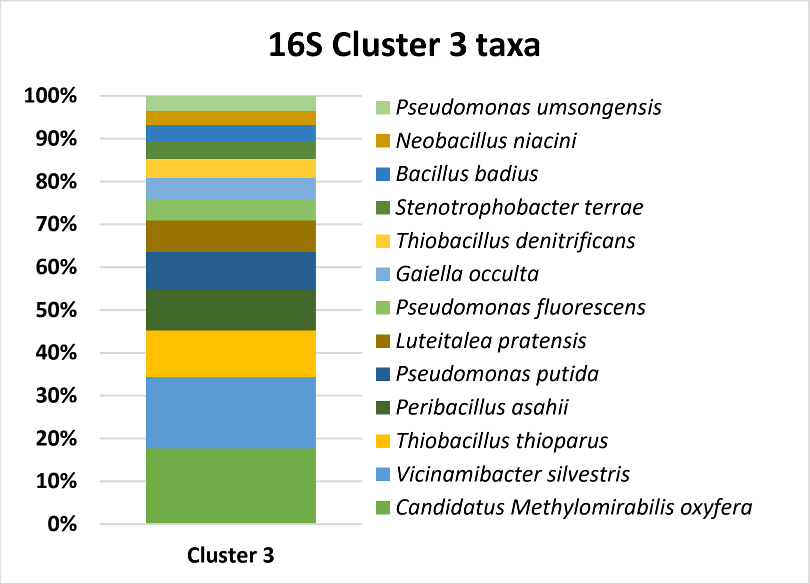

- Cluster 3 (C and C+O t0) is the most distinctive cluster: it is dominated by *Candidatus Methylomirabilis oxyfera* (linked to nitrite and methane in anoxic microenvironments), together with *Thiobacillus thioparus* and *T. denitrificans*. Opportunistic/PGPR such as *Pseudomonas putida*, *P. fluorescens*, *P. umsongensis*, and *Peribacillus asahii* are also abundant. This profile is consistent with higher soil moisture and micro-anoxia, availability of reactive nitrogen, and labile carbon inputs (organic residues).
